# iWhale: a computational pipeline based on Docker and SCons for detection and annotation of somatic variants in cancer WES data

**DOI:** 10.1093/bib/bbaa065

**Published:** 2020-05-20

**Authors:** Andrea Binatti, Silvia Bresolin, Stefania Bortoluzzi, Alessandro Coppe

**Keywords:** Docker, Cancer, Bioinformatics, Pipeline, Whole exome sequencing

## Abstract

Whole exome sequencing (WES) is a powerful approach for discovering sequence variants in cancer cells but its time effectiveness is limited by the complexity and issues of WES data analysis. Here we present iWhale, a customizable pipeline based on Docker and SCons, reliably detecting somatic variants by three complementary callers (MuTect2, Strelka2 and VarScan2). The results are combined to obtain a single variant call format file for each sample and variants are annotated by integrating a wide range of information extracted from several reference databases, ultimately allowing variant and gene prioritization according to different criteria. iWhale allows users to conduct a complex series of WES analyses with a powerful yet customizable and easy-to-use tool, running on most operating systems (macOs, GNU/Linux and Windows).

iWhale code is freely available at https://github.com/alexcoppe/iWhale and the docker image is downloadable from https://hub.docker.com/r/alexcoppe/iwhale.

## Introduction

Malignant transformation of cells is driven by many factors, including the development of somatic mutations that may affect signalling pathways which govern cell behavior and the expression of cancer hallmarks [1]. Both molecular and bioinformatics advancements are helping early diagnosis of the disease as well as the identification of better therapeutic strategies facilitating personalized medicine [2].

From a biological point of view, it is well known that not all somatic variants present in cancer cells share the same importance: mutations [3–6] providing a growth advantage during neoplastic transformation (and thus driving disease development), called ‘driver’ variants, coexist with many ‘less deleterious’ or neutral ones, which are called ‘passenger’ variants [3].

**Figure 1:**
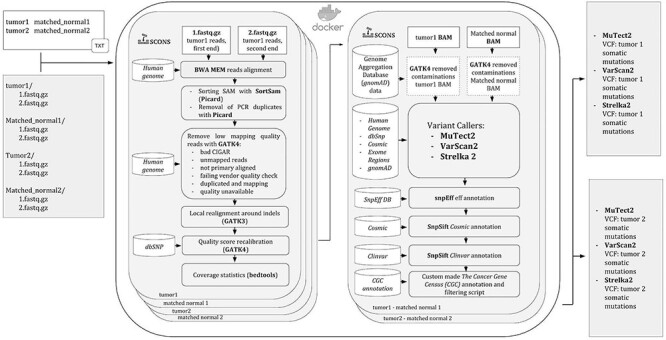
Flowchart diagram of the iWhale pipeline. The software runs under Docker and all the steps are managed by SCons.

Cancer exome sequencing is a popular and efficient way to get information about somatic mutations, which are not only one of the main causes of tumor development [7], but also of its aggressiveness [8] and progression [9]. Being the exome less than 3% of the human genome, the sequencing of exons is a timesaving and cheaper option than whole genome sequencing (WGS). In the last few years, the reduction in sequencing costs led to a substantial increase of the target region coverage, increasing the rate of detection of relevant mutations, also at subclonal level (i.e. those present only in a fraction of the considered cancer tissue sample under investigation). To distinguish somatic variants from germline and loss of heterozygosity variants, the exomes from tumor and control samples collected from the same patient are often compared. Despite the design of this analysis seems simple, a mere subtraction of control sample variants from tumor samples is not an appropriate strategy for the detection of somatic mutations. The steps used to detect somatic variants from matched tumor-control samples are complex and error prone due to several confounding factors, such as altered ploidy, intra-tumor heterogeneity and low tumor purity, and insertion of false positives or artifacts during tumor tissue conservation, library preparation, sequencing or reads alignment. Additionally, to perform the downstream analysis of the sequencing data, remarkable bioinformatics knowledge is required, including the installation and set up of several different software tools. To overcome these issues, pipelines for whole exome sequencing (WES) analyses have been developed in recent years. One of these tools is Fastq2vcf [10], a pipeline based on bash scripts, outputs not only the annotated variant call set for each caller, but also the consensus variant call set shared by different callers. Other Authors have tested and compared the performance of different variant callers from WES data. One of the first comparisons [11] was between GATK [12] and SAMtools [13], two of the first variant callers developed and still extremely used. Another available variant caller pipeline is SeqMule [14], which allows the user to obtain results of various alignment and analysis software. SeqMule consists of Perl scripts but all the external programs do need previous installation. From a biological point of view, it can be used for both Mendelian disease or cancer genome study. Another pipeline implemented in Perl is Cake, that integrates four variant callers and combines single nucleotide variants (SNVs) called by at least two software [15]. Other tools for WES include pipelines integrating multiple variant callers by machine learning approaches. For instance, NeoMutated pipeline [16] incorporates seven supervised machine learning algorithms to prioritize variants detected by seven callers. NeoMutate shows good performance compared to standard filtering protocols but unfortunately it cannot perform variant annotation; additionally, the pipeline is available only upon request. Another tool is SMuRF (Huang et al. 2019), an R package for the integration of results from different variant callers: it uses a supervised random forest approach, which however has been trained on WGS data, derived from only two tumor samples (from the International Cancer Genome Consortium). Finally, there are also web-based pipelines like Galaxy [18] and GotCloud [19]. The former is an open-source software that provides a very simple interface, which can be used to carry out WES analyses offering a good solution for developing easy usable applications. GotCloud [19] detects variants from large-scale sequencing data, performing various steps, starting with alignment, variant calling and quality score controls. It can be run on Amazon Web Services or in a local server, but its installation can be challenging for non-computational experts.

Overall, many of the above-cited software tools include command line pipelines which require to be installed on servers or local computers, very often presenting hard to solve dependencies. At the same time, even if user-friendly, web-based option exists, e.g. Galaxy, they come with some other significant drawbacks, such as the amount of data to be uploaded and the CPUs time to run the processes.

To overcome these limitations, and leveraging our experience in the field of WES applied to cancer research [8, 20–22], we present iWhale, an automated, easy-to-use and customizable software pipeline. It allows the identification of reliable and putative somatic SNVs and indels (insertions and deletions) in tumor samples (Figure 1) addressing the problems that most often come with the analyses of cancer WES data.

iWhale is based on Docker, a container platform to build and manage applications and launch them from macOs, Linux or Windows platforms. All steps and dependencies are controlled by SCons, automatically resuming the analysis from the last process run, in the event of any stop, like killing by error the process or computer shutting down.

The pipeline is made up of three different parts, in the first step reads are mapped to the reference human genome and alignments are optimized for variant calling. In this section of the pipeline, the software BWA [23], one of the most performing tools for exome and genome data alignment is employed [24]. The optimization of the alignments is performed by GATK4 and GATK3, which are software packages including several bioinformatics tools to manage WES and WGS data. The second part of iWhale consists of the variant detection step: here, the user can choose to employ (with default, or custom parameters) all or just some of three somatic variant callers chosen. We adopted variant callers Mutect2, Strelka2 and VarScan2 because they are specific for somatic variants and are based on different algorithms that complement each other. MuTect2 employs a haplotype-based strategy getting notably better detection of indels and structural variants with respect to position-based strategies. This tool locally assembles reads in a region and generates candidate haplotypes that may be represented by de Brujin-like graphs. Then, the likelihood of each haplotype is estimated by aligning reads to the haplotype and counting the read support. This approach is preferable in regions dense with variants, as it does not rely on local alignments, which are prone to errors, particularly in difficult regions. Strelka2 is a variant caller modeling joint allele frequencies and applying an additional random forest model trained on call-quality features. VarScan2 exploits a heuristic approach for the identification of potential somatic variants present in reads in accordance with algorithm-specific thresholds and then applies statistical tests or rules to call somatic variants. Although the user can choose to run only one of the variant caller methods, the union of variants detected by at least two methods is recommended to obtain robust results [12, 25]. Finally, iWhale annotates variants with SnpEff [26] and SnpSift [27] exploiting information from publicly available methods and databases. SnpEff is a genetic variant annotation and functional effect prediction toolbox, which annotates variants on transcripts and proteins. It also predicts the genomic region hit by the variant (i.e. coding, non-coding, regulatory region, splice site), codon changes and amino acid changes information, functional impact (missense variants, loss of function among others), epigenomic information, protein functional domains that could be affected by the variant among others. SnpSift associates variants with information about allele frequency on populations, the predicted impact on coded proteins or transcripts, the clinical significance and the known implication in genes dysfunction known to be cancer drivers. All this information is extremely useful for prioritizations of detected variants and interpretation of biological significance of mutated genes. Thus, users can run the software with almost no intervention, excluding a relatively simple data preparation, obtaining richly annotated variants that could help to understand what has generated the specific cancer in samples. Advanced users can launch personalized analyses adapting the parameters to the design and objectives of their specific research project.

At the light of all of the above considerations on currently available tools, i.e. that they tend to be difficult to set up and use, or, for the few user-friendly options, scarcely customizable and limited in range, a major advantage of iWhale is that it allows even non-expert users to conduct a complete, comprehensive WES cancer analysis, from reads mapping to somatic variants annotation.

## Implementation

iWhale is based on Docker (https://www.docker.com/), which is a container platform to build, manage and run applications from macOS, Linux or Windows.

All analysis steps are tied together by SCons (https://scons.org), a software construction tool written in Python (www.python.org) designed to facilitate software development by managing the building and compilation of large software projects specifying step dependencies so to ensure the correct workflow of the software. SCons automatically runs all the pipeline steps and, in case of a sudden interruption, is capable of resuming the analysis from the last successfully completed step of the process.

To run the pipeline, the user needs to download the Docker iWhale image from Docker hub (https://hub.docker.com/) with the Docker *pull* command. Notably, the design based on Docker allows running iWhale also leveraging parallel computing, if needed.

iWhale runs in a specific directory containing two directories for each matched sample (one for tumor and one for the corresponding control samples) including the two paired-end fastq files, a text file including the sample names and finally a Python file with changes in pipeline parameters, if any are specified. All parameters are optional except for the specification of the .bed file, which requires the user to specify the target exome regions (option exomeRegions). The software also needs the gziped version of the bed file made by bgzip and the .tbi index done with tabix [13]. Among the different parameters of the analysis that can be customized, one is particularly important, as the user can choose to employ all or a subset of the three variant callers included in the pipeline (additionally, with the possibility to change their default settings for each of them). All databases needed by iWhale should be inserted in a specific directory, which must be declared by the user in the Docker *run* command (i.e. the command that initiates the analysis, launched from the specified working directory).

## Methods

The human reference genome (GRCh37 and GRCh38) has been downloaded from Ensembl genome browser (ftp://ftp.ensembl.org/pub/grch37/current/fasta/homo_sapiens/dna/ and ftp://ftp.ensembl.org/pub/grch38/current/fasta/homo_sapiens/dna/) and Epstein-Barr virus (EBV) reference genome from https://www.ncbi.nlm.nih.gov/nuccore/AJ507799.2?report=fasta. All chromosomes and EBV sequences have been joined into a single FASTA file for the whole human genome. As required by BWA [23] for faster mapping of reads, the reference genome was indexed using Picard (http://broadinstitute.github.io/picard/) to obtain the .dict index, while samtools was used to create the .fai.

The pipeline workflow is divided into two main steps: after mapping of sequences to the human reference genome, somatic variants are called and annotated.

Initially, the paired-end fastq files obtained by WES are mapped to the reference genome by BWA MEM, which generates the BAM alignment files.

To improve alignment quality, various steps are executed after the first mapping phase: reads are sorted and PCR duplicated are flagged by Picard (http://broadinstitute.github.io/picard/). GATK4 [13] is used to remove low quality reads (CIGAR, i.e. spliced alignments, failing vendor quality check, duplicated) and reads with low mapping quality (unmapped, or where mapping quality is unavailable, or where reads are not primary aligned, i.e. mapping in different genome sites but with less mapping quality). Then, local realignment around indels and base quality score recalibration (BQSR) are performed by GATK3 [23, 28]. Local realignment around indels is performed to improve alignment quality in difficult regions. Genome aligners can only consider reads in an independent way resulting in many mismatches near the indels with respect to the reference genome, which ultimately can result in false SNPs. In addition, reads having an indel near their start or end are often incorrectly aligned. Local realignment takes into account all reads spanning a given position necessary to obtain alignments with higher scores to support indels. This refinement along with base quality recalibration, may reduce false positives resulting from stochastic and systemic sequencing and alignment errors. A previous study reported that BWA mapping generated misalignment for over 15% of the reads spanning known homozygous INDELs [29], leading to the call of false variants. It also tested the effect of local realignment around indels, showing a noticeable effect on indel calling at low coverage, and only a minor effect on detection of true variants by haplotype-based callers (e.g. MuTect2 and Strelka2). In any case, local realignment around indels was proven to improve true indel detection, particularly with specific variant callers [30] such as VarScan2.

Raw sequencing data scores are subject to various sources of non-random technical errors, which can result in over- or under-estimated base quality scores. Quality score recalibration (BQSR) is carried out with GATK4, obtaining the matched BAM files subsequently to be used for somatic variant calling.

Once the alignment of reads to the genome is refined, the calling and annotations of somatic variants are performed.

Variant calling can be done with all the possible combinations of three softwares: Mutect2 [13], VarScan2 [14] and Strelka2 [15]. Variant call format (VCFs) files are annotated by iWhale using a series of different databases. SnpEff [13,16] is used to annotate genomic variants and estimates the impact and/or deleteriousness of variants, also considering information about genomic location and changes induced on coded protein. ClinVar [10] which ‘aggregates information about genomic variation and its relationship to human health’ to associate variants to diseases is used to define known disease-associated variants. dbSNP [11, 18] is employed to annotate variants as likely neutral, or likely pathogenetic, as compared to known cases. COSMIC (cancer.sanger.ac.uk) [11] is used to identify mutations that were previously associated with cancer development. Finally, Genome Aggregation Database (gnomAD) [9, 11, 18] is used to retrieve information on variant allele frequencies, as calculated from 125 748 exomes and 15 708 genomes of unrelated individuals (sequenced in different population genetic and disease-specific studies).

All reference data employed in the annotation step of the pipeline, can be downloaded as gz or zip files from compgen site (http://compgen.bio.unipd.it/downloads/annotations.tar.gz, http://compgen.bio.unipd.it/downloads/annotations.zip), with the exception of COSMIC files, which need to be downloaded by the user from https://cancer.sanger.ac.uk/cosmic/download (non-academic users must sign up and logins).

At the end of the annotation process, the user is provided with two annotated VCFs, for SNVs and indels, for each sample analyzed.

## Evaluation with simulated data

We tested iWhale capability to detect somatic variants using simulated paired ‘tumor’ and control WES samples. Tumor samples were obtained by spiking variants in HG00246 and NA20505 WES samples obtained from the International Genome Sample Resource [31], which were used as controls (Table 1). After quality control with FASTQC [32] and adapter removal, iWhale aligns reads of control samples to the GRCh37 human genome. Next, 1619 and 786 randomly selected COSMIC v.90 variants (VAF range 0.02–0.6) were inserted by BamSurgeon [33] in HG00246 and NA20505 BAMs, respectively. The obtained BAMs were sorted by name using Samtools [13] and converted to paired end .fastq files by *bamtofastq* command of Bedtools [34].

**Table 1:** Results obtained by iWhale on simulated mutations obtained on samples HG00246 and NA20505 by BamSurgeon software

Sample ID	Project	Library type	Variants inserted	True positive	False positive	False negative
HG00246	Male (GBR population)	WES paired end	1619	1614	1	5
NA20505	Female (Toscani population)	WES paired end	786	785	1	1

**Figure 2:**
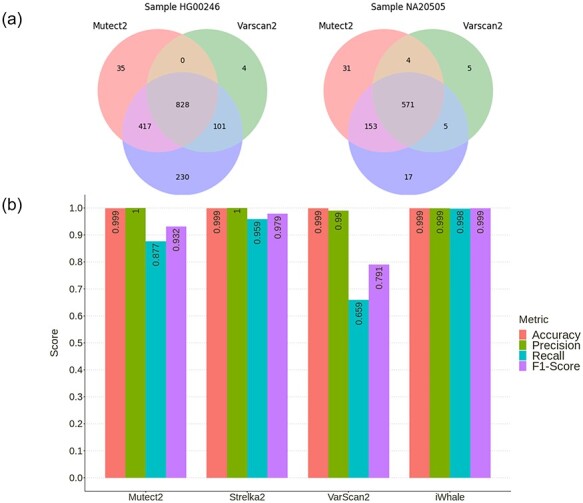
(**A**) Venn diagram displaying the comparison of the detected variants found by the three variant callers used by iWhale. (**B**) Performance evaluation of iWhale, using simulated paired tumor and control WES data.

iWhale was launched for each tumor-control matched sample using default settings and somatic variants detected in tumor samples were compared with spiked in variants. Detected true positive variants were 1280 and 798 by Mutect2, 1576 and 746 by Strelka2 and 932 and 584 by Varscan2 (Figure 2A). The results showed that the intersection of the three variant callers (828 in HG00246 and 571 in NA20505) would miss almost half (48.8%) of the variants in HG00246 and 27.4% of the variants in NA20505. Conversely, iWhale employing its default strategy based on the union of all three variant callers detected 1614 (99.7%) and 785 (99.9%) true positive variants. The false negatives were five in HG00246 sample while only one in NA20505 (Table 1) and only one false positive variant was detected in both samples by iWhale. High accuracy (0.99), precision (0.99), recall (0.998), F1-Score (0.999) and very low (0.001) false discovery rate were obtained with iWhale, run in default mode (Figure 2B). Despite the fact that each of the three variant callers showed high accuracy and precision, they provided inferior performances for recall and F1-Score if compared to iWhale (Figure 2B). In summary, running on simulated data with default settings, iWhale returned very good performance metrics.

### Sample analysis

To illustrate iWhale workflow with an actual, real-life sample analysis, the software was applied to the re-analysis of WES data of the tumor and control from a patient with Juvenile myelomonocytic leukemia [22]. These data were previously analyzed with UnifiedGenotyper and VariantFiltration (with default parameters) software from the GATK suite: a non-synonymous variant c.820G > A in *WAS* (WASP Actin Nucleation Promoting Factor) gene was identified [22]. This variant was validated and functionally studied, demonstrating that the alanine to threonine change in position 274 (p.Ala274Thr) destabilizes WASP auto-inhibition, potentially alters the protein localization and leads to its aberrant activation within the hematopoietic compartment [22].

The analysis of the same WES data with the iWhale pipeline (with all parameters set to default values), identified 24 416 somatic variants called by at least one method. MuTect2 and Strelka2 gave a relatively limited list of reliable somatic variants, while VarScan2 outputs a considerably larger list of candidates, which should be filtered (by considering caller-specific parameters), to reduce false positives. In our test, we kept VarScan2 variants if: supported by both strands with a minimum coverage of 20; supported by at least six reads for the alternative allele, presenting a minimum average base quality for variant-supporting reads of 30; satisfying a minimum variant allele frequency > 0.2 and a *P*-value threshold (for variants calling) less than 0.01. With these filters, we came up with 9726 reliable somatic variants, mostly ‘passenger.’ To identify the variants with a putative ‘driver’ role, only variants with a SnpEff predicted impact ‘HIGH’ or ‘MODERATE’ and with a cancer-specific FATHMM [35] ‘deleterious’ status were considered. Common somatic variants with a population allele frequency > 5% in gnomAD were filtered, obtaining 167 somatic SNVs and 24 indels. Notably, the previously validated pathogenetic *WAS* variant [22], described before, was included in this group. Moreover, other, previously undetected variants were identified by our method, including *NOTCH1* (c.1693G > A) and *ING1* (c.527C > T), highlighting iWhale discovery power. Both variants were tagged as potential ‘drivers’ by the Cancer Genome Interpreter webtool (https://www.cancergenomeinterpreter.org) [36].

*NOTCH1* c.1693G > A is a deleterious substitution (p.Val565Met) in the EGF domain of *NOTCH1. NOTCH1* encodes a transmembrane receptor and an important transcriptional regulator of importance for normal development of many tissues, including blood cells. NOTCH1 is a master regulator of T cell maturation [37]. It can act as both an oncogene and a tumor suppressor [31, 32] and was already found mutated in Juvenile myelomonocytic leukemia [38]. NOTCH1 is a master regulator of T cell maturation; besides its aberrant activation of *NOTCH1* is a hallmark of T cell acute lymphoblastic leukemia and it is found mutated in at least 65% of cases [39, 40]. Finally, NOTCH1 increases c-MYC expression to mediate the activation of pathways promoting leukemia cell growth and metabolism [41].

The *ING1* (c.527C > T, p.Pro176Leu) variant affects an epigenetic regulator, which modulates gene expression and cell growth, and with a previously reported tumor suppressor role [42, 43]. ING1 physically interacts with TP53 promoting cell growth arrest and apoptosis [43]. *ING1* gene expression has been found to be downregulated or lost in many cancer types, including childhood acute lymphoblastic leukemia [42, 44], and a loss-of-function mechanism could be speculated for the identified variant.

Therefore, iWhale, used with default settings, was able to detect already known and confirmed variants as well as other, likely to be relevant (i.e. as they hit regions with previously reported important roles in cancer); this suggests that our pipeline can provide a detailed landscape of somatic variants in cancer cells.

## Conclusion

iWhale is an automated and modular tool to detect and annotate somatic variants from WES data of cancer samples allowing the user to select and combine three variant callers for the analyses. The combination of variants detected by more than one method is recommended to obtain comprehensive results [6, 7], since different variant callers based on different theoretical premises can complement each other. Moreover, our pipeline creates richly annotated variant files, by using annotations derived from different databases, which thus facilitates the identification of ‘driver’ variants.

From a computational perspective, iWhale is a robust pipeline, and can be run in any platform that supports Docker. The defined dependencies between the various steps of the calculations and different intermediate level results files allow re-run the pipeline and restart the analyses from the last concluded step, if needed. The open source software is available on GitHub (github.com) and can be cloned and modified by any developer.

Notably, being a tool used by its developers, iWhale will be regularly updated and bug fixed. We envisage that with the feedback from users and developers, we will keep on improving the method, aiming to positively contribute the field by providing a user-friendly pipeline, allowing a broad range of scientists (i.e. including relatively non-expert computer users) to extract results from WES data, relevant for cancer studies.

Overall, iWhale makes it easy to perform a complex analysis of cancer WES data. It yields reliable results that could give an important contribution to address and understand cancer molecular foundations; this, in turn, should help the development of new therapeutic approaches and, in terms of personalized medicine, allow better responses to cancer treatment.

**Key Points**

- The pipeline performs cancer whole exome sequencing automatic data analysis in a user friendly way, returning reliable somatic variants in variant call format.
- Three variant callers are integrated (Mutect2, Strelka2 and VarScan2).
- ClinVar, Cosmic, dbSNP, The Cancer Gene Census and Genome Aggregation Database data are used for detailed annotation of identified variants.
- Docker technology allows easy installation and distribution of the software, making it easy to run on the most used operating system.
- SCons provides complete automation of the process and recovery, if needed.

## Notes

**Andrea Binatti** is a research fellow at the Department of Molecular Medicine at the University of Padua. His research is on development and application of bioinformatics methods for genomics data analysis of solid or blood cancer patients.

**Silvia Bresolin** is assistant professor at the Department of Women’s and Children’s Health of the University of Padova. Her field of interest is next generation sequencing and transcriptoma data generation and analysis in hematological pediatric malignancies.

**Stefania Bortoluzzi** is associate professor of Applied Biology at the Molecular Medicine Department of the University of Padova. She leads a computational genomics group committed to cancer research and teaches biology and bioinformatics.

**Alessandro Coppe** is an assistant professor at the Department of Women’s and Children’s Health at the University of Padua. His work focuses on the analysis of exome and genome sequences from blood cancers of children patients. The Department of Women’s and Children’s Health at the University of Padua carries out both basic and applied research that promotes the health of newborn babies, children and adolescents.
